# The Functions of PPARs in Aging and Longevity

**DOI:** 10.1155/2007/39654

**Published:** 2007-11-25

**Authors:** Adnan Erol

**Affiliations:** Department of Internal Medicine, Faculty of Medicine, Celal Bayar University,45020 Manisa, Turkey

## Abstract

Peroxisome proliferator-activated receptors (PPARs) are key regulators in various age-associated pathophysiological processes related to energy metabolism and oxidative stress. A progressive rise of oxidative stress and related inflammatory reaction appears the hallmarks of the aging process and many age-related diseases. PPARs are important redox-sensitive transcription factors and their dyregulated activations seem to be major culprits for these pathological processes. Drugs targeting PPARs activity are already in widespread clinical use; however, based on these concepts, this review highlights the understanding of the role of PPARs in aging and indicates the necessary particular attention for the potential therapeutic uses of current PPAR agonists in age-associated diseases.

## 1. INTRODUCTION

Why and how does aging occur? This is the most interesting scientific question. A large number of theories of aging have been proposed. However, any appropriate theory must explain four main characteristics of aging: it is progressive, endogenous, irreversible, and deleterious for the individual [1]. Ageing is associated with immunosenescence; decreased hormonal secretion, metabolism, lean body, and bone mass; and increased fat accretion. As a consequence, inflammatory diseases, dyslipidemia, atherosclerosis, obesity, and type 2 diabetes incidence increases with age [2]. Most of those prominent features of aging are related to peroxisome proliferator-activated receptors (PPARs) activity. Actually, the elderly population, particularly in the industrialized world, is exposed over its life span to an ever increasing number of agonists of PPARs in the form of various therapeutic agents and environmental pollutants through respiration, in food, and water [3]. Hence in this review, two important PPARs-oriented characteristic mechanisms in aging, increased oxidative stress and decreased fuel metabolism, will be introduced briefly and then the role of PPARs in these processes will be discussed.

## 2. OXIDATIVE STRESS IN AGING

One of the prevalent theories in the current literature revolves around free radicals as causal agents in the process of aging. A range of 1–3% of inspired oxygen was metabolized to superoxide and hydrogen peroxide. Free radical reactions are ubiquitous in living systems. Life is apparently evolved spontaneously from basic chemicals, formed by free radical reactions largely initiated by ionizing radiation from the sun, so that life span evolved in parallel with the ability of organisms to deal with damage from free radicals [4].

In 1956, Denham Harman was the first to propose the visionary “free radical theory” of aging [5]. This concept received strong support in the 1970s from voluminous, particularly from the mitochondrial studies. It was subsequently discovered that reactive oxygen species (ROS) contribute to the accumulation of oxidative damage to cellular constituents. There is indisputable evidence of the formation of a range of macromolecular oxidation products encompassing lipids, proteins, and nucleic acids, presumably arising from ROS activity. However, counterbalancing oxygen radical damage, there exists a wide range of enzyme repair and small molecule scavenging [6].

One of the several theories concerning ROS component that has been extensively studied is the mitochondrial theory of aging, which hypothesizes that mitochondria are the critical component in control of aging. It is proposed that electrons leaking from the electron transport chain (ETC) produce ROS and these molecules can then damage ETC components and mitochondrial DNA, leading to further increases in intracellular ROS levels and a decline in mitochondrial function. In support of a mitochondrial theory of aging, evidence suggests that mitochondrial DNA damage is increased with aging [7].

Mitochondria were brought to attention in aging biology due to (1) the central role of mitochondria in producing chemical energy (ATP) to meet cellular requirements, and (2) the declines of basal metabolic rate and of physical performance in energy-requiring tasks, which are characteristic of the aging process [8]. Hence, mitochondria are considered the pacemakers of tissue aging due to the continuous production of oxygen, nitrogen free radicals, and related reactive species; and due to the selective oxidative damage that leads to mitochondrial dysfunction. It is now clear that mammalian life span is negatively related to the mitochondrial production of oxidizing free radicals and the dysfunctional mitochondria that determine mitochondrial and cellular turnover [8].

The age-related oxidative damage is, in a first approximation, due to an increased rate of generation of oxidant. Caloric restriction (CR) is the most effective intervention known to delay aging and increase life span. There is now evidence indicating that CR acts by decreasing oxidative stress and damage. Mitochondrial function and CR are apparently related to decreased superoxide production [9]. CR, a potent experimental paradigm for the retardation of aging, has been shown to exhibit broad and effective antioxidant properties, and anti-inflammatory effect [10]. The beneficial diverse antiaging effects of CR are associated with altered metabolism, particularly, reduced metabolic rate and oxidative stress; decreased fat mass, body temperature, and fasting glucose; improved insulin sensitivity; and altered neuroendocrine and sympathetic system [11]. Moreover, the study results indicated that CR decreased the expression of PPAR*γ*, PPAR*α*, and PPAR*β*/*δ*, which would lead to downregulation of fat metabolism [12].

## 3. ENERGY METABOLISM IN AGING

Fatty acids are a major substrate for oxidative energy metabolism in muscle, and indeed 60–90% of total oxygen consumption may be used to oxidize fatty acids. Fatty acids are oxidized in preference to carbohydrates. Oxidation of fatty acids or ketone bodies inhibits the utilization of both extracellular glucose and tissue glycogen [13]. Long-chain fatty acids (LCFAs) are the major component of dietary lipids, and carnitine palmitoyl transferase-1 (CPT-1) is a rate-limiting enzyme for *β*-oxidation of LCFA in mitochondria [14]. Reduced fatty acid oxidation and/or CPT-1 cause accumulation of triglyceride in tissue and are associated with many metabolic disorders [15].

Aging causes many changes in the energy metabolism of the muscle, particularly heart muscle. The aging myocardium shifts its fuel preference away from fatty acid oxidation (FAO) toward carbohydrate oxidation, which is a reversion to the neonatal state [16]. It was shown that mitochondria isolated from the hearts of senescent rats show a 20% lower rate of LCFA oxidation than mitochondria from young adult animals do. Conceivably, the overall rate of energy transduction by the mitochondria might be decreased, placing a limitation on the intensity of performance of mechanical work [17]. Interestingly, there is no evidence to suggest that the CPT-1 is the site of age-linked lesion suggesting CPT-1 activity is not changed with age. In stead, diminished mitochondrial content seems responsible for the reduction in FAO [18]. This change in fuel preference is typically paralleled by a reorganization of mitochondrial metabolism, including a shift in the gene expression and relative activities of FAO enzymes. This switch in fuel preference is thought to be maladaptive and contribute to the contractile dysfunction [19].

Aging is associated with increased body fat especially in the visceral area and peripheral tissues such as liver and skeletal muscle, and might be responsible for the age-associated increased risk for type 2 diabetes, hypertension, and atherosclerosis [20]. Recent studies have demonstrated a strong relationship between aging-associated reductions in mitochondrial function, dysregulated intracellular lipid metabolism, and insulin resistance. Petersen et al. showed that the older subjects were markedly resistant to insulin, as determined by the reduced insulin stimulation of muscle glucose metabolism [21]. Insulin resistance in the skeletal muscle of elderly is a linked defect to mitochondrial oxidative phosphorylation. However, further researches did not show changes in mitochondrial energy coupling [22]. This suggested an age-related reduction of mitochondrial number and/or function as the most likely culprit to explain the deficient energy metabolism of aging.

## 4. PPARs—AN OUTLINE

PPAR*α*, *δ*, and *γ* are nuclear transcription factors to the nuclear hormone receptor superfamily that controls key genes involved in energy homeostasis [23]. Consequently, PPARs play major roles in a broad spectrum of biological processes, including cell proliferation, differentiation, glucose homeostasis, eicosanoid signaling, insulin sensitivity, glucose and lipid metabolism, bone formation, and tissue remodeling [24]. A thorough examination of the biology of the PPARs is beyond the scope of this review; the reader is referred to more specialized reviews on this topic in [25–27].

PPARs require retinoid X receptors to form heterodimers, which allow binding to their specific PPAR-response elements (PPREs). Much is known about the activation of PPARs via PPREs and in most cases this leads to transcriptional activation of the target genes ([25] for detailed review), but far less information exists for the mode of PPAR modulation during aging. However, an age-related reduction of PPAR*α* in both mRNA and nuclear protein levels was shown [28].

What is the significance of multiple PPAR isoforms with distinct expression patterns? One possibility is that the three isoforms have different ligand specificities. Thus, the ratio of these receptors and their ligands provide a means of determining tissue-specific expression of target genes. In addition to their differential responsiveness to peroxisome proliferators, the three PPAR isoforms also display distinct yet overlapping expression patterns. The ratio of the PPAR isoforms, however, is likely to play a critical role in establishing the degree of responsiveness of tissues to specific activators [29].

There is evidence that PPAR*α* plays multiple roles in the aging process. PPAR*α* may influence aging through regulation of damage and repair processes after exposure to endogenous or environmental stressors. Corton et al. showed that PPAR*α* reduces the severity and frequency of a subset of age-dependent lesions in the mouse. These findings were in the liver, the kidney, and the heart, in which PPAR*α* is normally expressed, mediates regulation of lipid metabolizing genes, and regulates genes involved in maintaining the integrity of these tissues [30]. However, in the aged liver, a decrease in hepatic antioxidant activity, coupled with a PPAR*α* agonist-induced increase in liver oxidative stress and antiapoptotic effect of PPAR*α* agonists may expose these livers to the point of tumor formation [31].

Genetically long-lived mice are good animal models to estimate the importance of PPARs in longevity. Growth hormone receptor/binding protein knockout (GHR-KO) mice are characterized by markedly extended lifespan in comparison to normal controls. Interestingly, in contrast to GH-deficient and GH-resistant mutants, GH antagonist transgenic mice showed striking differences for several parameters including longevity. These mice are GH resistant or insensitive, have greatly reduced plasma IGF-I and insulin levels, and have low glucose level [32]. There are numerous indications that insulin, insulin release, and insulin actions play a major role in the control of mammalian aging. A recent report of increased life span in transgenic Klotho mice that are insulin-resistant raises the interesting possibility that aging can be delayed by reduced strength of the insulin signal, regardless of its underlying causes [33]. It has been hypothesized that insulin resistance is a physiological protection mechanism against aging and age-related disorders [34].

GHR-KO mice have significantly elevated PPAR*γ*, mRNA, and protein level in the liver. However, PPAR*γ* protein level in skeletal muscle was decreased in comparison to normal controls. The increased level of PPAR*α* in the liver of these mice suggests an increased FAO, which could be beneficial for insulin sensitivity. Similarly, PPAR*α* level in the muscle was decreased in GHR-KO animals. PPAR*β*/*δ* proteins were downregulated in the liver and skeletal muscle of KO mice ([35] and references therein).

As noted above, much of the accumulated evidence strongly implicates oxidative stress as a major factor contributing to the aging process and age-related diseases. Recent molecular studies show that the induction of oxidative stress is associated with the downregulation of PPARs [28]. More interestingly, the possible inverse association between age-related changes in PPARs levels and oxidative stress was further supported by the antioxidative action of CR. The results showed the antioxidative effects of CR blunt age induced decreases in PPAR*α*, PPAR*γ*, and mRNA levels; and in protein expression. Data further revealed that CR improved the age-induced decrease in PPAR DNA binding activity to PPRE. Also, CR induced prevention of the age-related decline in PPARs may partly reason in the suppression of the age-induced increased inflammation [28].

## 5. PPARs AND INFLAMMATION IN AGING

**Figure 1:**
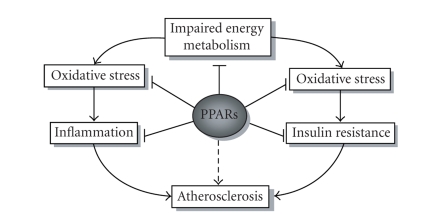
The main pathophysiological processes and their interactions leading to aging are depicted. The most likely inhibiting points of PPARs are indicated as possible targets for pharmacological treatment. Controversial issue of PPARs on atherosclerosis is marked by broken arrow.

The free radical theory of aging serves to explain not only basic mechanisms of aging but also the pathogenesis of a range of disease processes that consistently accompany aging, including atherosclerosis, other cardiovascular disorders, dementia, diabetes, arthritis, and osteoporosis [36]. In evaluating the free radical theory and its possible link to numerous age-related maladies, investigators have focused attention on the possibility that an increase in ROS, along with a concomitant disruption in redox balance, leads to a state of chronic inflammation (see Figure 1) [37].

Inflammation is a primary defense against threats to homeostasis. With aging, inflammatory responses may be overactive or even cause damage, resulting in adverse pathological conditions [38]. Oxygen and nitrogen-derived reactive species coupled with a deficient antioxidant defense capacity cause the redox imbalance. This oxidative stress-induced redox imbalance, in turn, activates many of redox-sensitive transcription factors, which generates various proinflammatory molecules [39]. Evidence also implicates the involvement of PPARs at the transcriptional level in the expression of proinflammatory mediators such as, nuclear factor-*κ*B (NF-*κ*B), signal transducers and activators of transcription (STAT)-1, and activating protein-1 (AP-1) [40]. In addition, several studies have demonstrated that PPAR*α* and PPAR*γ* inhibit the expression of inflammatory genes, such as cytokines, metalloproteases, and acute phase proteins [41].

Premature and enhanced age-dependent rise of oxidative stress and NF-*κ*B activation in the PPAR*α* knockout mice have been reported [42]. Ligand-activated PPAR*α* can suppress inflammatory reactions by inhibiting NF-*κ*B function. Enhanced IL-6 production by activated NF-*κ*B has been implicated in many pathophysiological dysfunctions of aging ranging from Alzheimer's disease to atherosclerosis [43]. However, intriguingly, high doses of the PPAR*α* subtype-specific ligand cause a marked activation of NF-*κ*B, whereas low or therapeutic doses of the hypolipidemic agents cause decreased NF-*κ*B activation, IL-6 production, and lipid peroxidation [42].

All major vascular and inflammatory cells express PPAR*γ*, including vascular smooth muscle cells, endothelial cells, and macrophages [44]. PPAR*γ* is expressed in monocytes and upregulated during their differentiation into macrophages [45]. Furthermore, PPAR*γ* ligands inhibit the production of proinflammatory cytokines, TNF*α*, and IL-1 [46]. These studies along with the observation of decreased carotid intima-media thickness in diabetic patients treated with the PPAR*γ* agonist troglitazone [47] suggest that PPAR*γ* activation might prevent atherogenesis. These vasculoprotective effects of PPAR*γ* agonists in humans may be attributed to their effects on inflammation [48]. On the other hand, PPAR*γ* activation induces expression of the scavenger receptor CD36, thereby promoting oxidized low density lipoprotein uptake and foam cell formation, predicting that PPAR*γ* is predominantly proatherogenic [43]. In addition, very recent meta-analysis, paradoxically, showed a significant increase in the risk of myocardial infarction and an increase in cardiovascular death of borderline significance with rosiglitazone, a member of PPAR*γ* agonists that is widely used as antidiabetic agent [49]. These confusing results, obviously, will create a lot of trouble for the clinical use of the agent, and further studies are required to define how much of these effects are the result of PPAR*γ*'s beneficial systemic metabolic effects versus its vascular and immune effects [50].

## 6. PPARs AND FUEL METABOLISM

Aged mammals, including humans, show a decreased capacity for FAO [51]. This may be an underlying cause of age-related decrease in energy metabolism, and increase in dyslipidemia. PPAR*α* is one of the primary metabolic nuclear receptors that act as sensors of fatty acid and other metabolites to enable the organism to adapt quickly to environmental changes by inducing or inhibiting appropriate metabolic genes and pathways [52].

PPAR*α* is expressed in metabolically active tissues including the liver, heart, kidney, and skeletal muscle [53]. Fatty acids are the primary natural ligands of PPAR*α*, which activates genes for fatty acid uptake and oxidative catabolism [54]. Fibrates are one of the synthetic ligands for PPAR*α*, and glucocorticoids induce PPAR*α* in response to stress [55] indicating that situations of stress can enhance the impact of nutritional factors on metabolic processes.

Data indicate a more general role for the major importance of PPAR*α* in the regulation of a mitochondrial oxidative enzyme gene because the mitochondrial FAO pathway is the primary source of cellular energy in many tissues [56]. However, PPAR*α* and *β*/*δ* are known to have overlapping roles in activating FAO as demonstrated through the use of PPAR*α* null mice. There were no obvious differences in FAO capacity, and the response to exercise was comparable between skeletal muscle of PPAR*α* null and wild-type mice [57]. In addition, the PPAR*α* agonist fenofibrate markedly increased fatty acid oxidation by the liver, but not by the skeletal muscle [58]. Furthermore, muscle from transgenic mice with a constitutively activated form of PPAR*β*/*δ* in skeletal muscle was composed of larger proportion of high oxidative capacity type I muscle fibres [59]. This led the investigators to conclude that PPAR*β*/*δ* is abundantly present in skeletal muscle, and skeletal muscle PPAR*β*/*δ* largely compensates for the lack of PPAR*α* in the null mice. However, it is unclear whether a crosstalk exists between these two nuclear receptors and whether they regulate cardiac FAO homeostasis independently. More importantly, PPAR*β*/*δ* was relatively unaffected by aging [57]. The actions of PPAR*β*/*δ* in skeletal muscle, that is, oxidative myofiber remodeling and increase of fatty acid burning capacity [60], may give hope to the specific agonists of this nuclear receptor for therapeutic usefulness in the age-related metabolic diseases.

In both cardiac and skeletal muscle all three isoforms in the following ranking order: PPAR*β*/*δ* *>* PPAR*α* *>* PPAR*γ* could be detected. PPAR*γ* expression is confined to a limited number of tissues, primarily adipose tissue, and vascular cells [61]. The expression of PPAR*γ* in muscle cells has been controversial issue, if present at all, the amount of PPAR*γ* in these cells is probably low [62]. The enhanced expression of PPAR*γ* within skeletal muscle cell under certain conditions may transdifferentiate into adipocyte-like cells [63]. Concerning the importance of age-associated sarcopenia, the use of medicine forcing PPAR*γ* activation needs much more attention in aged people.

## 7. PPARs AND METABOLIC SYNDROME IN AGED PATIENTS

Aging has been associated with a reduced capacity for oxidative phosphorylation in muscle, most likely due to a decline in mitochondrial content and/or function [64]. Given the strong evidence linking mitochondrial dysfunction with aging, insulin resistance, and type 2 diabetes [22], it is important to determine the significance of the clinical interventions with PPAR agonists. Furthermore, the clustering of these metabolic risk factors termed metabolic syndrome, which consequently increases the likelihood of atherosclerotic cardiovascular diseases, tends to increase with aging (see Figure 1) [65].

It is well established that insulin sensitivity normally declines with age in humans [66]. Thiazolidinedione (TZD) compounds are a new class of insulin-sensitizing drugs [67], which improve insulin sensitivity, glucose tolerance, and the lipidemic profile in type 2 diabetes, as well as in obese nondiabetic subjects [68]. It is demonstrated that PPAR*γ* expression is not changed in old rats. The administration of rosiglitazone restores practically all changes in lipid and glucose metabolism introduced by old age [69]. More importantly, according to results of Diabetes Reduction Assessment with Ramipril and Rosiglitazone Medication (DREAM) study, rosiglitazone substantially reduces incident of type 2 diabetes and increases the likelihood of regression to normoglycaemia in adults with impaired fasting glucose, impaired glucose tolerance, or both [70].

Dramatic changes in fat depot size, fat tissue distribution, and function occur throughout life span [71]. Although total body fat may decrease in old age, percent body fat declines very little, and may even remain constant or increase. This occurs because fat is redistributed from fat depots to other sites. This decline in fat within traditional fat depots in old age is accompanied by accumulation of fat in muscle, bone marrow, and other sites outside fat depots. Reasonable explanation for these significant age-induced fat depot changes came from the study showing that old animal preadipocytes expressed less PPAR*γ*. Hence, preadipocyte fatty acid handling changes with aging, with increased susceptibility to lipotoxicity and impaired fatty acid induced adipogenesis. This, in turn, may ultimately contribute to fat tissue dysfunction, compromising capacity of fat tissue to protect other tissues from lipid accumulation and lipotoxicity [72]. Consequently, paradoxical lipotoxicty of fat cell progenitors potentially reasons in systemic metabolic consequences.

Intriguingly, PPAR*γ* +/− mice displayed greater insulin sensitivity than their wild-type (WT) littermates did [73]. Further research, however, showed that this effect occurred only in aged animals. In other words, the PPAR*γ* receptor deficient animals are relatively protected from the normal physiological age-induced decrease in insulin sensitivity [74]. Thus therapeutic maneuvers that could produce relative PPAR*γ* deficiency might be of clinical value for the treatment of insulin resistance in old age [73].

Concerning the ageing process, PPAR*γ* may perhaps be thought of a “harmful” receptor, due to the combination of its role in the thrifty response and the increased caloric intake seen in modern “Western diets [75]. At this point, PPAR*γ* can be divided agonists into two groups, classical full agonists, which are represented by the TZDs, such as rosiglitazone and pioglitazone, and newer partial agonists, which were developed, in large part, to reduce the side effect of weight gain observed with full agonists. Partial agonists are compounds that produce activity below that of saturating concentrations of a full agonist [50]. Partial agonism might be an interesting approach to the therapy of type 2 diabetes, particularly in elderly, because partial or minimal PPAR*γ* activation may have beneficial effects by stimulating insulin sensitivity without promoting adipogenesis.

**Figure 2:**
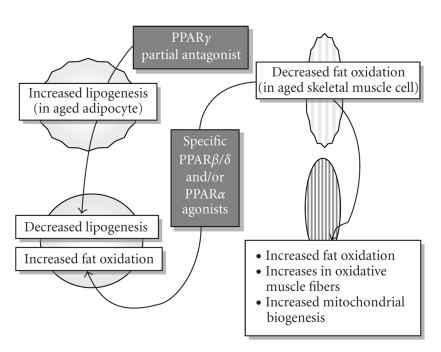
In aging organism, two important sites regarding the energy metabolism, adipocyte with the increased lipogenesis, and skeletal muscle with the decreased fat oxidation are shown. Possible future therapeutic fields and expected beneficial alterations are also indicated.

Reduction of PPAR*γ* activity by small-molecule partial antagonists improves the metabolic profile in mice. Although still at a preliminary stage, several compounds have been identified that may prove efficacious in the treatment of the metabolic syndrome and type 2 diabetes [50, 75]. One of these new generation compounds could prevent adipogenesis, insulin resistance, and obesity by blocking endogenous ligand-induced activation of PPAR*γ*. In addition, these compounds may increase glucose disposal in peripheral tissues (see Figure 2) [76].

Another concern of PPAR*γ* activation by TZD use in the elderly is bone fracture. PPAR*γ* activation by TZD may also affect bone through an increase in bone marrow adiposity and a decrease in osteoblastogenesis, resulting in reduced bone formation [77, 78]. Since PPAR*γ* expression increases in marrow mesenchimal cell with aging, PPAR*γ* activation by rosiglitazone resulted in distinctive changes in bone microarchitecture and strength [79]. Additionally, TZDs affect aromatase pathway, leading to decreased estrogen production with an increase in bone resorption [80]. More importantly, DREAM [70] and ADOPT [81] trials clearly showed the impact of TZDs on bone fracture risk in older women.

Old age is associated with dyslipidemia, obesity, and type 2 diabetes mellitus, which are the risk factors, increase the major cardiovascular events in late life [82]. These alterations in lipid metabolism are partially related to a profound reduction in the liver expression and activity of PPAR*α*, and several of its target genes, in old animals. Therefore, these animals may become resistant to the hypotriglyceridemic effects of fibrate class of hypolipidemic drugs, which are PPAR*α* ligands [83]. This may provide an explanation for the disappointing primary outcome results of the large-scaled FIELD study, because the ages of the patients in this study significantly were in old-age group [84].

Fibrates and statins are the two most commonly used hypolipidemic drugs. Statins mainly reduce intrahepatocyte cholesterol levels through the inhibition of hepatic 3-hydroxy 3-methylglutaryl-CoA reductase. Interestingly, it was demonstrated that atorvastatin is a synthetic statin reversed the age-related reduction of hepatic PPAR*α*. However, atorvastatin administration prevents the age-related metabolic changes associated with the reduction in hepatic PPAR*α* expression and activity only in old male rats, senescent females being practically unresponsive to the drug [85].

## 8. CALORIC RESTRICTION, LONGEVITY, AND PPARs

Increasing evidence demonstrates that CR profoundly affects the physiological and pathophysiological alterations associated with aging and markedly increases life span in several species including mammals [35]. Recent studies on CR provide clear evidence that CR likely exerts its diverse benefits through its antioxidative ability to suppress age-related oxidative stress while upholding the antioxidant defense system [9, 10].

CR is also known to alter expression of large number of genes involved in lipid metabolism and insulin signaling. Expression of many of the same genes is regulated by PPARs acting as transcription factors. This suggests a possibility that PPARs mediate the effects of CR [35]. In a recent study, the effect of 2,4-thiazolidinedione, starting material of the various synthetic TZD, compared with the short-term CR on the aging process. Study results showed that PPAR*γ* activation suppresses age-induced inflammation and oxidative stress via the down-regulation of NF-*κ*B. It has been proposed that the anti-inflammatory and antioxidative properties of PPAR*γ* activators may be potential effective therapeutic approaches to the treatment and/or prevention of age-induced inflammatory diseases (see Figure 1) [86].

Dwarf mice, which exhibit decreased levels of insulin, insulin like growth factor (IGF)-I, and increased insulin sensitivity, have defects in growth hormone action and share a number of beneficial phenotypic characteristics with rodents on CR diets. These shared decreases in insulin and IGF-I may be the basis for beneficial phenotypic effects in common with CR and dwarf mutations. Like CR animals, dwarf mice are protected from spontaneous and chemically induced cancer, age-dependent declines in immune function, and collagen cross-linking ([87] and references therein). Gene array studies indicated that the genes regulated by PPAR*α* were either upregulated in dwarf mice or their expression increased in response to PPAR*α* agonist treatment, which was interpreted as some of the beneficial effects associated with the dwarf phenotype that may be caused by constitutive activation of PPAR*α* and regulated genes [88].

PPAR*α* seems to play roles in long-term CR. The expression of the PPAR*α* gene but not protein was increased in the livers of CR animals [89]. PPAR*α*-deficient mice develop oxidative stress at an earlier age than aged-matched wild-type mice. In addition to roles in lipid metabolism, PPAR*α* likely carries out other unexpected functions during CR. PPAR*α* was partially or completely required for CR to downregulate acute phase genes responsive to inflammatory cytokines, possibly through the negative influence of PPAR*α* on the important inflammatory genes [87]. PPAR*α* activators diminish tissue lipid peroxide levels, abrogate age-associated constitutively active NF-*κ*B, and reduce spontaneous cytokine production in aged wild-type but not in PPAR*α*-deficient mice [90]. PPAR*α* agonist action on oxidative status might also contribute to lower redox activation of transcription factors, such as NF-*κ*B and AP-1 [86].

Corton and Brown-Borg found significant overlaps between the CR transcript profiles in wild-type mice and the profiles altered by agonists of PPAR*α* and hypothesized that some effects of CR are mediated by PPAR*α* [87]. However, activation of PPAR*α* also regulates the genes in gluconeogenesis in addition to its well-known effects on the genes of fatty acid oxidation [87]. Therefore, impaired glucose excursion via PPAR*α* agonist administration in elderly people who are not in calorie restricted diet increases the formation of ROS and induces hyperinsulinemia, which enhances the steady-state level of oxidative stress [34]. Masoro hypothesized that the lifetime maintenance of reduced levels of blood glucose and insulin without compromising glucose fuel use partly contributes to the antiaging actions of CR [91]. Thus the identification of higher affinity and selective PPAR*α* ligands particularly for skeletal muscle and adipose tissue not for liver, and the administration in the early periods might slow aging and prevent age-associated metabolic disorders (see Figure 2) [92].

A central question is whether the benefits of CR are a passive result of lower caloric intake or the consequence of an active regulatory program. Molecular and genetic studies in model organisms have suggested that CR may be a regulated process, with the silent information regulator 2 (SIR 2) gene playing an important role. Mammalian ortholog of this gene, SIRT1, mediates a broad array of physiological effects that occur in animals on a CR diet [93]. SIRT1 is widely distributed in mammalian tissues and partners with different effectors [87]. One of them is PPAR*γ*, which is repressed by SIRT1 activation. SIRT1 activity is predicted to counter PPAR*γ*-mediated adipogenesis [94]. Given the impact of SIRT1 on PPAR*γ* activity and because PPAR*γ* activity helps determine age-related insulin resistance [95], SIRT1 may have an important role in metabolic diseases and link the effects of food consumption to body fat mass and diseases of aging [95]. Until now there is no experimental evidence that SIRT1 represents the molecular link between reduced adiposity and increased lifespan. Considering the insulin sensitizing effects of PPAR*γ* agonists, it is at least controversial whether inhibition of PPAR*γ* would be beneficial to human longevity [96]; but, it may remain an experimental support for the above-mentioned harmful effects of PPAR*γ* agonists. It is also interesting to speculate that the increased frequency of atherosclerosis in old-age people could be a PPAR*γ*-driven maladapted macrophage response that occurs when the stimulation of the innate immune response and cholesterol efflux are overwhelmed by the proatherogenic effects [50, 75].

## 9. CONCLUSIONS

Aging is a complex stochastic process determined by genetic and environmental factors. Similarly, the activity and functions of PPARs in aging are highly confusing.

1. Moderate levels of PPAR*γ* activation are beneficial because efficient energy conservation and storage have allowed survival through periods of food shortages. However, affluent lifestyles of elderly population in industrialized world already exposed them to natural PPAR*γ* ligands throwing this tightly regulated system into a metabolic turmoil, so-called metabolic syndrome.
2. PPAR*γ* signaling plays an important role in the pathophysiology of human age-related osteoporosis by increasing the number of adipocytes in bone marrow and decreasing the number of osteoblasts. Recent DREAM study clearly indicated this adverse affect of the PPAR*γ* agonist, rosiglitazone. Similarly, chronic administration of TZDs was accompanied by fat accumulation in the bone marrow cavity and impaired hematopoiesis, resulting in anemia [97].
3. Collectively, modulating or clearly inhibiting PPAR*γ* activity in elderly, rather than activating it, might be the preferred therapeutic strategy as an antiaging approach (see Figure 2) and also to treat age-associated metabolic disorders including obesity, type 2 diabetes and cardiovascular diseases.
4. Recent data link adipose tissue, nutrition, and xenephormesis as pivotally involved in the processes of aging and longevity [98, 99]. Resveratrol, a polyphenolic compound present in red grape and wine, stimulates SIRT1 expression that extends life expectancy in treated animals via CR mimetic activity [95, 99]. Upon food withdrawal Sirt1 protein represses and binds to genes controlled by the fat regulator PPAR*γ* (96).
5. Age-induced decrease in PPAR*α* expression may reveal tissues unprotected against acute or chronic stress. Therefore, it should be aimed pharmacologically to increase the aging-induced decrease of protein expression of PPAR*α* because PPAR*α* may play roles in stress resistance and/or longevity. Currently, unique proven strategy, which improves age-induced downregulation in PPAR*α* molecular system, is an exercise training [100]. Once again, this finding underlines the importance of lifestyle modification in aged people. The administration of current pharmacological ligands of PPAR*α* such as fibrates to geriatric patients may not work expectedly because of the deficiency of their receptors.
6. There is no change in the expression of PPAR*β*/*δ* in aging cells. Physical exercise simulating actions of PPAR*β*/*δ* on skeletal muscle seems to be PPAR*β*/*δ* agonists that might be expected agents for the metabolic disorders, particularly, of the age- associated (see Figure 2). The outcome of ongoing clinical trails for the therapeutic usefulness of these agents will provide eagerly awaited results in the very near future.
